# Complementary prognostic value of stress perfusion imaging and global coronary flow reserve derived from cardiovascular magnetic resonance: a long-term cohort study

**DOI:** 10.1186/s12968-023-00930-3

**Published:** 2023-03-16

**Authors:** Satoshi Nakamura, Masaki Ishida, Kei Nakata, Masafumi Takafuji, Shiro Nakamori, Tairo Kurita, Haruno Ito, Kaoru Dohi, Hajime Sakuma

**Affiliations:** 1 Department of Radiology, Mie University Hospital, 2-174 Edobashi, Tsu, Mie 514-8507 Japan; 2 Department of Cardiology and Nephrology, Mie University Hospital, Tsu, Mie Japan

**Keywords:** Coronary flow reserve, Coronary sinus, Cardiovascular magnetic resonance, Myocardial ischemia, Long-term prognostic value

## Abstract

**Background:**

Phase-contrast cine cardiovascular magnetic resonance (CMR) quantifies global coronary flow reserve (CFR) by measuring blood flow in the coronary sinus (CS), allowing assessment of the entire coronary circulation. However, the complementary prognostic value of stress perfusion CMR and global CFR in long-term follow-up has yet to be investigated. This study aimed to investigate the complementary prognostic value of stress myocardial perfusion imaging and global CFR derived from CMR in patients with suspected or known coronary artery disease (CAD) during long-term follow-up.

**Methods:**

Participants comprised 933 patients with suspected or known CAD who underwent comprehensive CMR. Major adverse cardiac events (MACE) comprised cardiac death, non-fatal myocardial infarction, unstable angina, hospitalization for heart failure, stroke, ventricular arrhythmia, and late revascularization.

**Results:**

During follow-up (median, 5.3 years), there were 223 MACE. Kaplan–Meier curve analysis revealed a significant difference in event-free survival among tertile groups for global CFR (log-rank, p < 0.001) and between patients with and without ischemia (p < 0.001). The combination of stress perfusion CMR and global CFR enhanced risk stratification (p < 0.001 for overall), and prognoses were comparable between the subgroup with ischemia and no impaired CFR and the subgroup with no ischemia and impaired CFR (p = 0.731). Multivariate Cox proportional hazard regression analysis showed that impaired CFR remained a significant predictor for MACE (hazard ratio, 1.6; p = 0.002) when adjusted for coronary risk factors and CMR predictors, including ischemia. The addition of impaired CFR to coronary risk factors and ischemia significantly increased the global chi-square value from 88 to 109 (p < 0.001). Continuous net reclassification improvement and integrated discrimination with the addition of global CFR to coronary risk factors plus ischemia improved to 0.352 (p < 0.001) and 0.017 (p < 0.001), respectively.

**Conclusions:**

During long-term follow-up, stress perfusion CMR and global CFR derived from CS flow measurement provided complementary prognostic value for prediction of cardiovascular events. Microvascular dysfunction or diffuse atherosclerosis as shown by impaired global CFR may play a role as important as that of ischemia due to epicardial coronary stenosis in the risk stratification of CAD patients.

**Supplementary Information:**

The online version contains supplementary material available at 10.1186/s12968-023-00930-3.

## Background

Coronary artery disease (CAD) is one of the major causes of death worldwide. Management of CAD has evolved primarily around the detection and treatment of significant stenoses in the epicardial coronary arteries. Stress perfusion cardiovascular magnetic resonance (CMR) is useful for detecting regional myocardial ischemia caused by luminal stenosis in the epicardial coronary arteries. However, qualitative stress perfusion CMR evaluates relative changes in myocardial perfusion and has limited value in detecting diffuse, subclinical atherosclerosis or microvascular dysfunction. Quantitative perfusion CMR has recently shown promising results in diagnosis of obstructive CAD and microvascular dysfunction [1, 2] and prediction of prognosis [3], but qualitative perfusion method may still be more dominant in clinical settings. Phase-contrast cine CMR quantifies blood flow in the coronary sinus (CS), which represents approximately 96% of the total blood flow in the myocardium [4]. The measurement of CS blood flow by phase-contrast cine CMR during stress and at rest provides a global coronary flow reserve (CFR), representing the ratio of hyperemic total coronary flow to baseline total coronary flow. Through the quantitative assessment of total coronary blood flow, global CFR could complement the qualitative and relative approach of stress perfusion CMR. A recent study showed the usefulness of combining stress perfusion CMR and global CFR by CS flow measurement for detecting flow-limiting CAD as determined by fractional flow reserve [5].

Several studies have investigated the prognostic value of global CFR by the CS flow measurement [6–9]. Kato et al. demonstrated the usefulness of CS-derived global CFR for predicting cardiac events in patients with suspected or known CAD during follow-up (median, 2.3 years) and showed that CS-derived CFR and stress perfusion CMR had comparable prognostic value [6]. Indorkar et al. revealed that impairment of CS-derived global CFR offered an independent predictor when adjusted for CMR predictors including myocardial ischemia over a median follow-up of 2.1 years [7]. Nevertheless, the long-term prognostic value of CS-derived global CFR remains unknown. How agreement or disagreement between abnormal CFR and ischemia affect the prognosis of CAD patients is also unclear. The aim of this study was therefore to evaluate the long-term prognostic value of CS-derived global CFR in patients with suspected or known CAD and to investigate whether global CFR has an additional and complementary prognostic value to stress perfusion CMR.

## Methods

### Study population

A total of 1366 consecutive patients ≥ 45 years old with suspected or known CAD who underwent comprehensive CMR between January 2009 and December 2015 at our hospital were considered for participation in this study. Known CAD was defined as having a history of coronary revascularization or myocardial infarction (MI). Exclusion criteria were: (1) non-ischemic cardiomyopathy; (2) valvular disease; (3) congenital heart disease; (4) non-analyzable images in phase-contrast cine CMR; or (5) early revascularization within 90 days after CMR. The institutional review board approved this retrospective study and waived the need to obtain individual consent.

### Image acquisition

All CMR images were acquired using a 1.5T or 3T scanner (Achieva 1.5T/Achieva 3T; Philips Healthcare, the Netherlands). A 32 element cardiac-surface coil wase used for radiofrequency reception. Each CMR examination consisted of cine CMR, stress and rest perfusion CMR, stress and rest phase-contrast cine CMR of the CS, and late gadolinium enhancement (LGE) CMR. To assess left ventricular (LV) volumes, systolic function and LV mass, breath-holding cine CMR was acquired with a segmented balanced steady-state-free precession (bSSFP) sequence in the short-axis planes covering the entire LV. Scan parameters for the 1.5T scanner were: slice thickness, 10 mm; cardiac phases, 20; repetition time (TR), 3.2 ms; echo time (TE), 1.6 ms; flip angle (FA), 55°; field of view (FOV), 35 × 35 cm; acquisition matrix, 192 × 192; and reconstruction matrix, 256 × 256. Scan parameters for the 3T scanner were: TR, 2.8 ms; TE, 1.4 ms; FA, 55°; FOV, 35 × 35 cm; acquisition matrix, 176 × 308; and reconstruction matrix, 352 × 352.

Stress perfusion CMR consists of 4 or 3 short-axis slices acquired every 2 cardiac cycles or every cardiac cycle with a saturation-recovery balanced bSSFP or turbo field echo (TFE) sequence for 1.5T scanner (TR, 3.0 ms; TE, 1.5 ms; FA, 40°; FOV, 36 × 32 cm; acquisition matrix, 192 × 153; SENSE factor, 2; time between saturation preparation pulse and center of k-space acquisition, 200 ms; duration of image data acquisition, 211 ms; slice thickness section, 8 mm) or 3T scanner (TR, 2.9 ms; TE, 1.4 ms; FA, 20°; FOV, 32 × 29 cm; acquisition matrix, 224 × 131; SENSE factor, 2.5; time between saturation preparation pulse and center of k-space acquisition, 110 ms; duration of image data acquisition, 141 ms; slice thickness section, 10 mm). For stress perfusion CMR, dynamic imaging was initiated 3 min after starting adenosine triphosphate infusion (160 mg/kg/min) and continued for 1 min. Gadoterate meglumine (Gd-DOTA; Guerbet Japan, Tokyo, Japan) was injected at a dose of 0.03 mmol/kg and a flow rate of 4 mL/s, followed by a 20-mL saline flush for stress perfusion CMR. Rest perfusion CMR was performed with an identical set-up at 10 min following stress perfusion CMR.

**Fig. 1:**
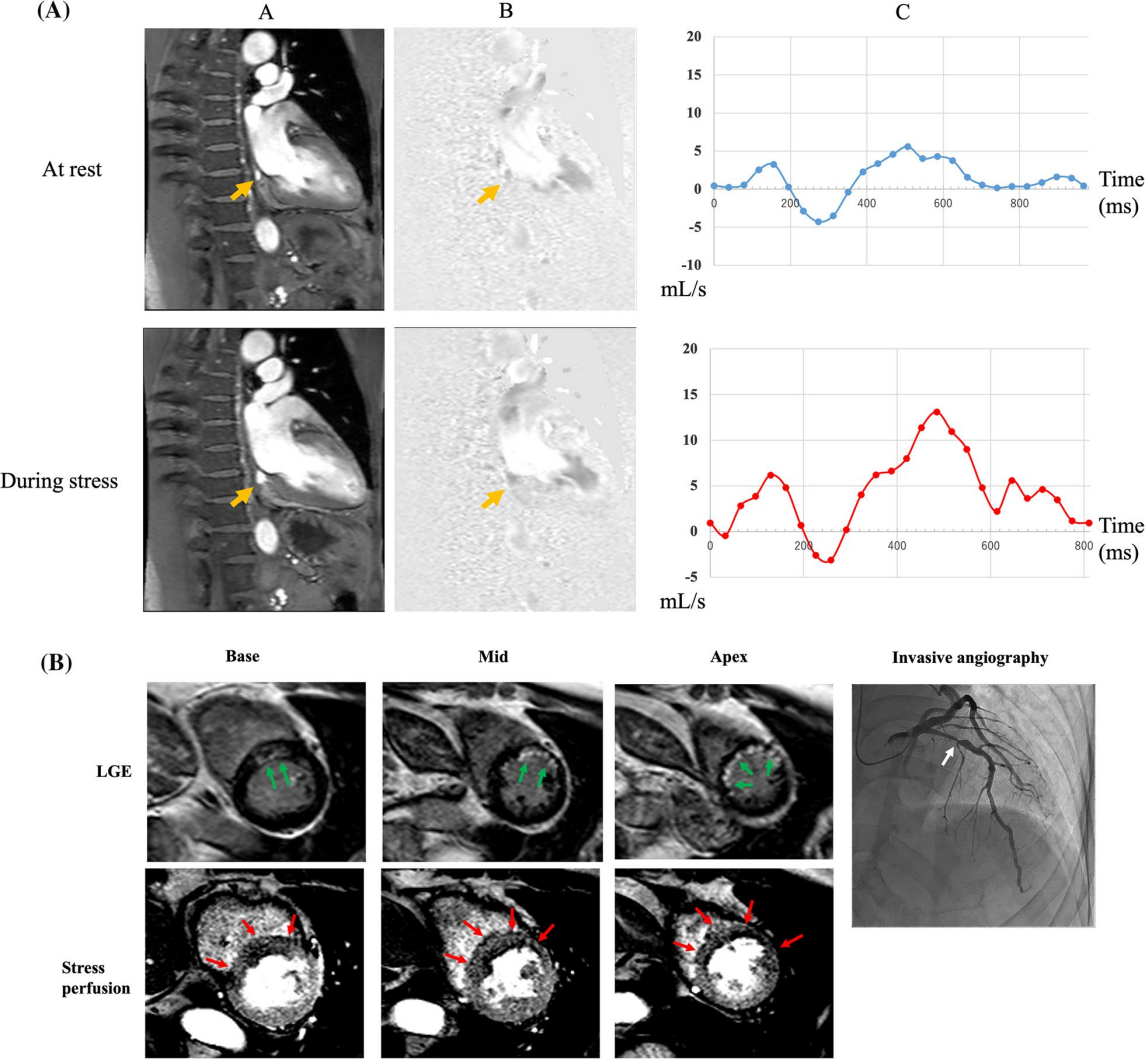
**A** An example for measurement of blood flow in the coronary sinus. A 67-year female presented with chest pain and underwent phase-contrast cine cardiovascular magnetic resonance (CMR) performed at a 3T scanner including magnitude image (**A**), phase-difference image (**B**), and blood flow curve in the coronary sinus (CS; arrows) at 1 cardiac cycle (**C**). Rest and stress coronary blood flow were 75 mL/min and 303 mL/min, resulting in coronary flow reserve (CFR) of 4.0, which was in the higher tertile. Stress perfusion CMR and late gadolinium enhancement (LGE) did not show ischemia and infarction. CMR results of the patient were diagnosed to be normal. **B** A representative case for stress perfusion CMR. A 76-year male with hypertension and dyslipidemia underwent stress perfusion CMR and LGE at a 1.5T scanner. LGE images showed sub-endocardial infarction in the antero-septal wall of the left ventricle (LV; green arrows). Stress perfusion CMR showed transmural hypoperfusion area suggesting ischemia in the septal to anterior walls of the LV (red arrows). Invasive angiography demonstrated significant stenosis in the proximal left anterior descending artery

CS blood flow during stress and at rest were measured by breath-hold phase-contrast cine CMR for 1.5T scanner (TR, 8.6 ms; TE, 5.6 ms; FA, 15°; FOV, 36 × 26 cm; acquisition matrix, 192 × 112; 16 phases per cardiac cycle; and velocity encoding, ± 70 cm/s) or 3T scanner (TR, 4.8 ms; TE, 3.1 ms; FA, 10°; FOV, 25 × 21 cm; acquisition matrix, 176 × 97; 25 phases per cardiac cycle; and velocity encoding, ± 80 cm/s) as shown in Fig. 1A. Stress and rest phase-contrast cine CMR images were acquired during breath-holding with shallow inspiration (15 s) on an imaging plane perpendicular to the CS immediately after stress and rest perfusion CMR [7]. The imaging position was carefully chosen so that blood flow in the CS was measured on the slice as close as possible to the orifice to the right atrium and the CS was visible throughout the cardiac cycle.

LGE CMR was acquired in the same LV short-axis planes as used for cine CMR using a three-dimensional inversion recovery TFE sequence 5–10 min after intravenous administration of Gd-DOTA with a cumulative dose of 0.15 mmol/kg for 1.5T scanner (TR, 3.8 ms; TE, 1.2 ms; FA, 15°; FOV, 40 × 36 × 5 cm; acquisition matrix, 224 × 156 × 5; reconstructed matrix, 256 × 256 × 10; SENSE factor, 2; TFE factor, 24) or 3T scanner (TR, 4.6 ms; TE, 2.2 ms; FA, 15°; FOV, 38 × 34 × 5 cm; acquisition matrix, 240 × 192 × 5; reconstructed matrix, 384 × 384 × 10; SENSE factor, 3; TFE factor, 33).

### Image analysis

CMR images were analyzed using dedicated software (cvi^42^; Circle Cardiovascular Imaging Inc., Calgary, Canada). At end-diastole and end-systole, endocardial LV borders were manually traced in contiguous short-axis cine CMR images covering the apex to mitral valve plane to calculate LV end-diastolic volume (LVEDV) and LV end-systolic volume (LVESV) and ejection fraction (LVEF). After tracing epicardial LV borders at end-diastole, LV mass was calculated as the sum of the myocardial volume at end-diastole multiplied by the specific gravity (1.05 g/mL) of myocardial tissue.

Two independent observers (one with 20 years and one with 8 years of experience in CMR), blinded to clinical information and other imaging results, evaluated stress perfusion CMR and LGE CMR (Fig. 1B). Disagreement between the two observers was settled by a consensus reading. Perfusion defects were defined as hypo-enhanced regions that persisted for at least three phases after peak contrast enhancement and followed a coronary distribution. Inducible ischemia was defined as a stress perfusion defect in a segment without LGE or larger than LGE. Myocardial ischemia was defined when inducible ischemia was present in > 10% of LV myocardium, namely in ≥ 3 of 32 subsegments (endocardial and epicardial sectors for each of the 16 segments) [10]. On LGE images, presence or absence of LGE was visually assessed in 16 segments.

CS blood flow (mL/min) was calculated by integrating the product of cross-sectional area and mean velocity in the CS from the 16 images across the cardiac cycle. Global CFR was defined as CS blood flow during stress divided by CS blood flow at rest. Contours of the CS were manually traced on magnitude images at each cine frame. The traced region of interest was applied on corresponding phase-difference image, and cross-sectional area and mean velocity were recorded. Impairment of global CFR was defined as showing a lower tertile in global CFR.

### Follow-up

Follow-up information was collected through a review of hospital records or telephone interviews with the interviewer blinded to CMR results between 2019 and 2020. If the same patient had more than one CMR between 2009 and 2015, the first CMR was used for this cohort study and the patient was followed up from that time. Major adverse cardiac events (MACE) comprised cardiovascular death, non-fatal MI, unstable angina, hospitalization for heart failure, stroke, ventricular arrhythmia and late (> 90 days after CMR) revascularization. Severe events were defined as MACE other than late revascularization. Hard events were defined as cardiovascular death and non-fatal MI. Cardiovascular death was defined as death caused by acute MI, ventricular arrhythmias, heart failure or stroke. Non-fatal MI was defined as prolonged angina accompanied by new electrocardiographic (ECG) abnormalities and increased cardiac biomarkers. Unstable angina was defined as new-onset, worsening, or rest angina requiring hospital admission.

### Statistical analysis

Continuous variables are presented as the mean ± standard deviation and categorical variables are expressed as frequency (percentage). Univariate Cox proportional hazards regression analysis was performed to identify potential predictors of MACE among coronary risk factors and CMR imaging parameters and multivariate Cox proportional hazards regression analysis was performed using the “enter” method for variables from univariate analysis showing values of p < 0.05 to determine independent predictors of MACE These Cox analyses were performed in all patients and subsets of patients with known/suspected CAD. The results are reported as hazard ratios (HRs) with 95% confidence intervals (CIs). The incremental value of a predictor over another predictor was evaluated by calculating global chi-square values. Net reclassification improvement (NRI) with the addition of global CFR to coronary risk factors plus ischemia was calculated, and continuous NRI and integrated discrimination improvement were estimated. Kaplan–Meier curves were used to estimate event-free rates for MACE, severe events or hard events in all patients and subsets of patients with known/suspected CAD or with 1.5T or 3T CMR and differences between time-to-event curves were compared using the log-rank test. Annualized event rates were calculated by dividing 5-year Kaplan–Meier event rates by 5. Interobserver agreement was evaluated using Bland–Altman analysis, intraclass correlation coefficient (ICC), and quadratic-weighted kappa analysis by two observers (one with 8 years and one with 7 years of experience in CMR) in a subset of 40 randomly selected patients. Two-sided p-values < 0.05 were considered statistically significant. All analyses were performed using the SPSS (version 23.0; Statistical Package for the Social Sciences, International Business Machines, Inc., Armonk, New York, USA) and the R statistical package (version 3.4.4, R Foundation for Statistical Computing, Vienna, Austria).

## Results

Of the 1366 patients ≥ 45 years old who underwent CMR, this study excluded 372 patients with non-ischemic cardiomyopathy (n = 128), valvular disease (n = 12), congenital disease (n = 8), non-analyzable images (n = 52) or early revascularization (n = 172). As 61 patients were lost to follow-up, the final study population comprised the remaining 933 patients with suspected (n = 596) or known (n = 337) CAD. Table 1 shows baseline patient characteristics in the study population. Results for CMR are presented in Table 2. Ischemia was observed in 270 patients (29%), and global CFR was 3.3 ± 1.6 (mL/min).

**Table 1:** Patient characteristics

Characteristic	All patients (n = 933)
Male	618 (66)
Age (mean ± SD)	68 ± 9
Body mass index (mean ± SD)	24 ± 4
Coronary risk factors	
Hypertension	630 (68)
Dyslipidemia	554 (60)
Diabetes	304 (33)
Current smoker	103 (11)
Family history of CAD	146 (16)
Known CAD	337 (36)
History of myocardial infarction	211 (23)
Prior coronary revascularization	302 (32)

Except where indicated, data are numbers of patients (percentages)

CAD: coronary artery disease

**Table 2:** Imaging results

Parameters	
Heart rate, beats/min (mean ± SD)	68 ± 11
LVEDV index, mL/m^2^ (mean ± SD)	80 ± 24
LVESV index, mL/m^2^ (mean ± SD)	36 ± 21
LVEF, % (mean ± SD)	58 ± 11
LVEF < 50%	185 (20)
CS blood flow during stress, mL/min (mean ± SD)	222 ± 125
CS blood flow at rest, mL/min (mean ± SD)	76 ± 58
Global CFR (mean ± SD)	3.3 ± 1.6
Ischemia	270 (29)
LGE	359 (39)

Except where indicated, data are numbers of patients (percentages)

LVEF: left ventricular ejection fraction; LVEDV: left ventricular end-diastolic volume; LVESV: left ventricular end-systolic volume; CAD: coronary artery disease; CS: coronary sinus; CFR: coronary flow reserve; LGE: late gadolinium enhancement

**Fig. 2:**
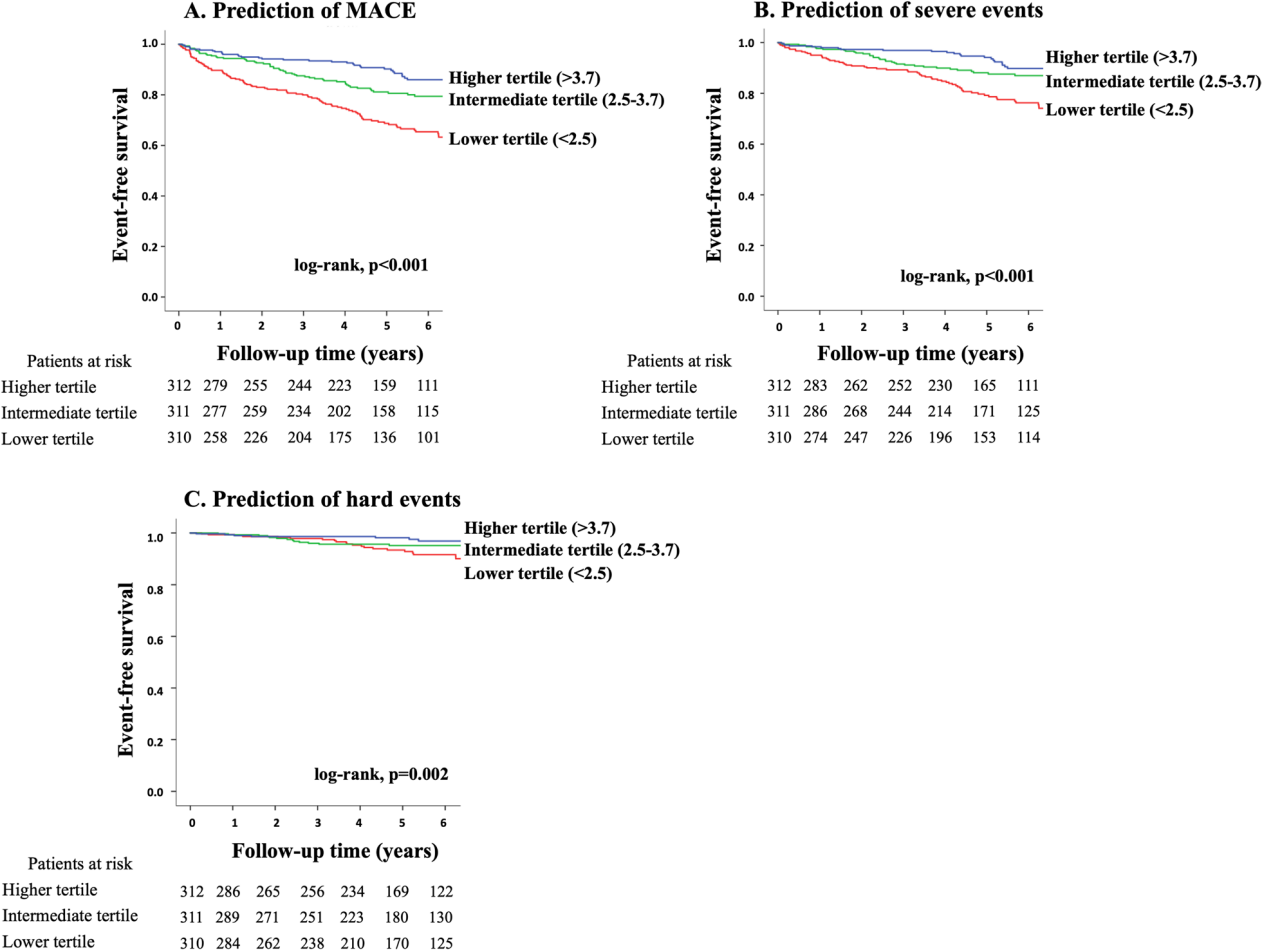
Long-term risk stratification by global CFR. Annualized event rates in patients with higher, intermediate and lower tertiles for global CFR were 2.0%, 3.8% and 6.3%, respectively, for prediction of major adverse cardiovascular event (MACE) (log-rank, p < 0.001; **A**), 1.2%, 2.4% and 4.2%, respectively, for prediction of severe events (p < 0.001; **B**), and 0.4%, 1.0% and 1.3%, respectively, for prediction of hard events (p = 0.002; **C**)

**Fig. 3:**
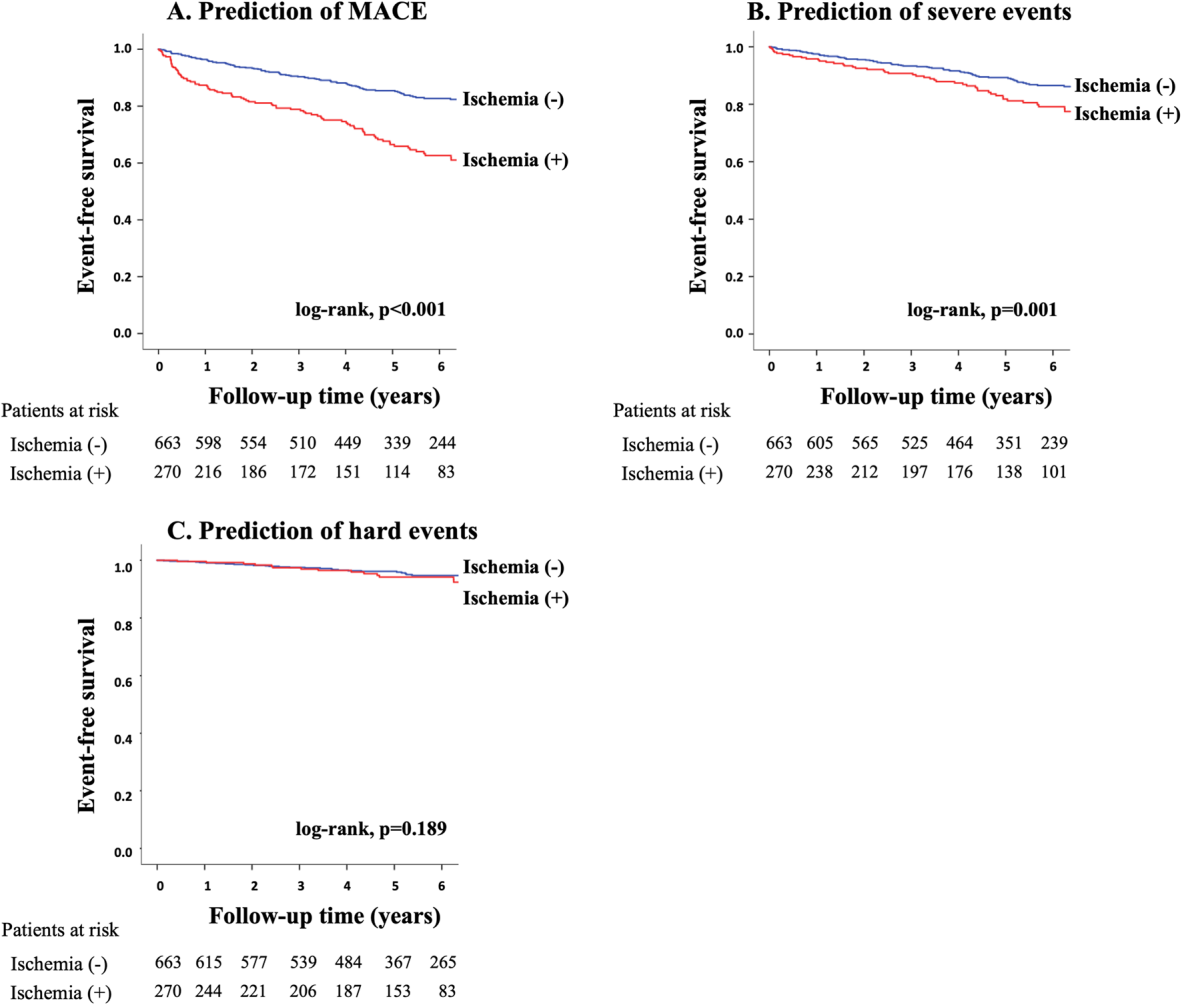
Long-term risk stratification by stress perfusion CMR. Annualized event rates in patients with and without ischemia were 6.7% and 2.9%, respectively, for prediction of MACE (log-rank, p < 0.001; **A**), 3.6% and 2.1%, respectively, for prediction of severe events (p = 0.001; **B**), and 1.2% and 0.8%, respectively, for prediction of hard events (p = 0.189; **C**)

**Fig. 4:**
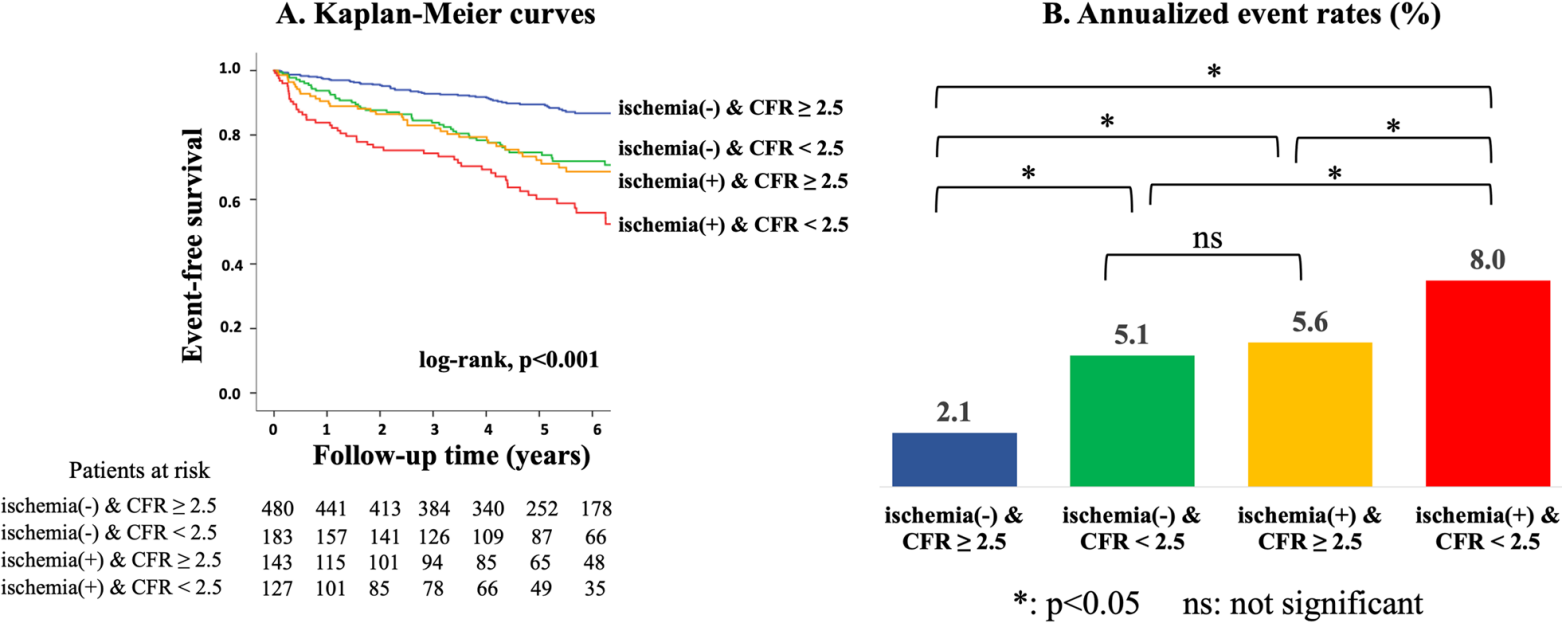
Risk stratification by combination of global CFR and stress perfusion CMR. **A** Risk stratification by combining stress perfusion CMR and global CFR (p < 0.001 for overall). **B** Annualized event rates in subgroups. A subgroup with ischemia and impaired CFR (CFR < 2.5) experienced a significantly higher annualized event rate of 8.0% compared with the other subgroups (with ischemia and no impaired CFR: event rate 5.6%, p = 0.012; with impaired CFR and no ischemia: event rate 5.1%, p = 0.002; and without ischemia or impaired CFR: event rate 2.1%, p < 0.001). The subgroup with ischemia and no impaired CFR and the subgroup with no ischemia and impaired CFR had comparable prognosis (p = 0.731), while both of these subgroups experienced worse prognosis (p < 0.001, respectively) than in the absence of ischemia and impaired CFR

**Fig. 5:**
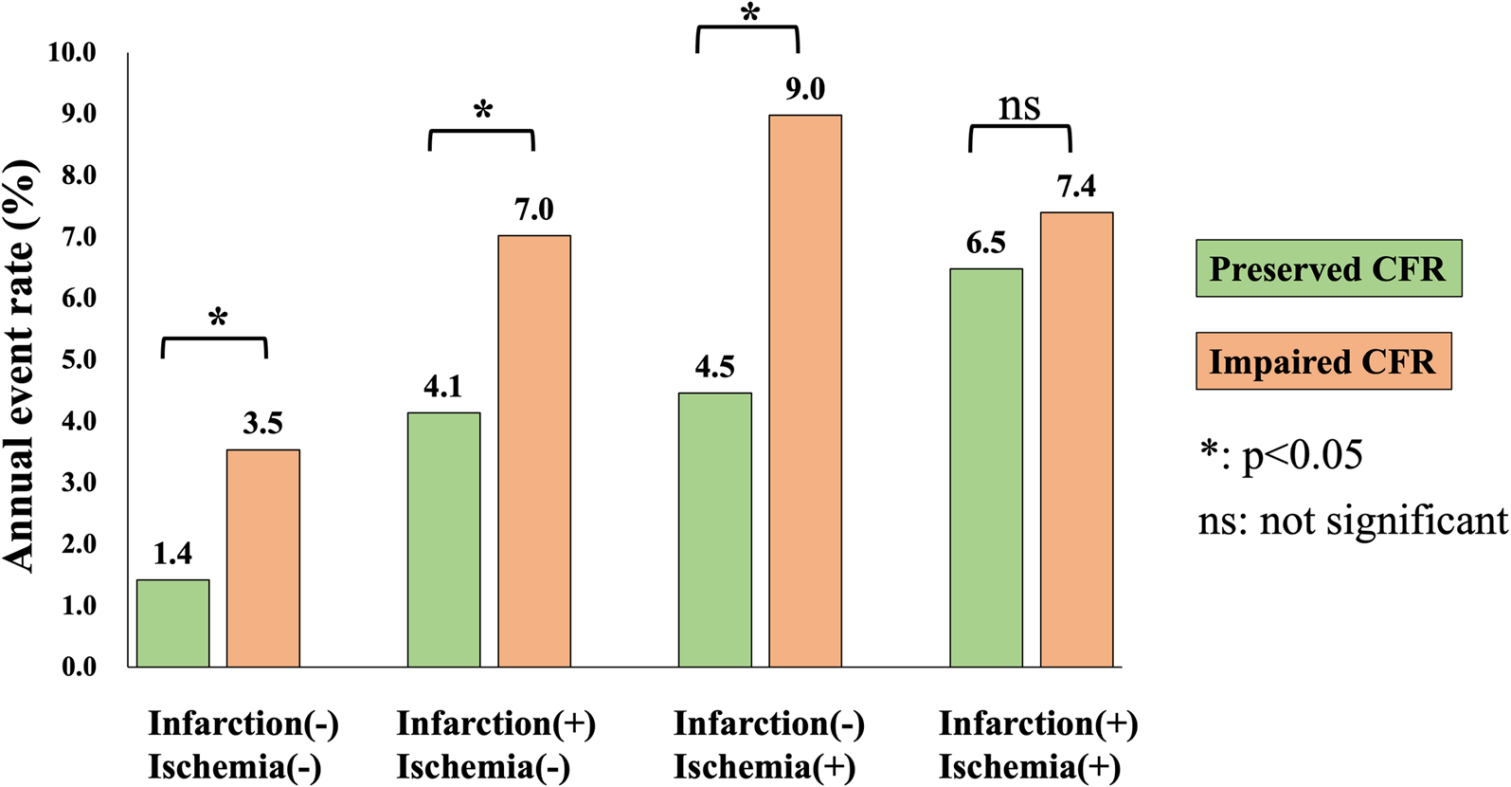
Risk stratification of global CFR in subgroups stratified by ischemia/infarction. The prognostic value of global CFR in subgroups stratified according to the presence/absence of ischemia and infarction is shown. Annualized event rates for patients with preserved or impaired CFR were 1.4% and 3.5%, respectively, in those without ischemia or infarction (log-rank, p = 0.009), 4.1% and 7.0%, respectively, in those with infarction and no ischemia (p = 0.007), 4.5% and 9.0%, respectively, in those with ischemia and no infarction (p = 0.028), and 6.5% and 7.4%, respectively, in those with both ischemia and infarction (p = 0.450)

During follow-up (median, 5.3 years), MACE was observed in 223 patients (cardiovascular death [n = 39], non-fatal MI [n = 13], unstable angina [n = 29], heart failure [n = 40], stroke [n = 27], ventricular arrhythmia [n = 4] and late revascularization [n = 71]). Kaplan–Meier curve analysis revealed a significant difference in event-free survival among patients with lower (< 2.5), intermediate (2.5–3.7) or higher (> 3.7) tertiles in global CFR for prediction of MACE (log-rank, p < 0.001; Fig. 2A), severe events (p < 0.001; Fig. 2B), and hard events (p = 0.002; Fig. 2C). There was a significant difference in event-free survival between patients stratified by the presence or absence of ischemia for prediction of MACE (p < 0.001; Fig. 3A), severe events (p = 0.001; Fig. 3B), but not hard events (p = 0.189; Fig. 3C). Figure 4 illustrates risk stratification using a combination of stress perfusion CMR and global CFR (p < 0.001 for overall), and the subgroup with ischemia and no impaired CFR and the subgroup with no ischemia and impaired CFR showed comparable prognoses (p = 0.731), while a significant difference in event-free survival was found in each comparison (p < 0.05, respectively) between any two remaining subgroups stratified by combination of ischemia and impaired CFR. Figure 5 shows the prognostic value of global CFR among subgroups stratified according to the presence/absence of ischemia and infarction. Impairment of CFR was associated with unfavorable outcomes in patients without ischemia or MI, with MI and no ischemia, and with ischemia and no MI (p < 0.05 each), while no significant difference in event-free survival was seen among patients with ischemia and MI.

In patients with known CAD, Kaplan–Meier curve (Additional file 1: Fig. S1) analysis showed a significant difference in event-free survival among patients stratified by global CFR tertiles for prediction of MACE (log-rank, p = 0.017), whereas there was not a significant difference in event-free survival among patients stratified by ischemia or combination of impaired CFR and ischemia. In patients with suspected CAD, Kaplan–Meier curve (Additional file 1: Fig. S2) analysis demonstrated a significant difference in event-free survival for prediction of MACE among patients stratified by global CFR tertiles (log-rank, p < 0.001), ischemia (p < 0.001) or combination of impaired CFR and ischemia (p < 0.001). Furthermore, when the patients were divided into those who underwent 1.5T or 3T, Kaplan–Meier curve analysis demonstrated a significant difference in event-free survival for prediction of MACE among patients stratified by global CFR tertiles (log-rank, p < 0.001), ischemia (p < 0.001) or combination of impaired CFR and ischemia (p < 0.001) both at 1.5T (Additional file 1: Fig. S3) and 3T (Additional file 1: Fig. S4).

Results for univariate Cox proportional hazard regression analysis for MACE are listed in Table 3, where CAD risk factors excluding family history of CAD were identified as significant predictors for MACE. Reduced LVEF, impaired global CFR, ischemia, and prior MI were significant predictors for MACE, and HRs for those imaging predictors were 2.4 (95% CI 1.8–3.2; p < 0.001), 2.3 (95% CI 1.7–3.0; p < 0.001), 2.4 (95% CI 1.8–3.2; p < 0.001), and 2.6 (95% CI 2.0–3.5; p < 0.001), respectively. Multivariate Cox proportional hazard regression analysis (Table 4) showed that impaired CFR remained an independent predictor (adjusted HR, 1.6 [95% CI 1.2–2.1]; p = 0.001) when adjusted for CAD risk factors (male, age, hypertensin, dyslipidemia, diabetes, and smoking), reduced LVEF, ischemia, and prior MI. In patients with known CAD, multivariate Cox proportional hazard regression analysis (Additional file 1: Table S1) showed that impaired CFR remained an independent predictor (adjusted HR, 1.5 [95% CI 1.0–2.1]; p = 0.048). In patients with suspected CAD, multivariate Cox proportional hazard regression analysis (Additional file 1: Table S2) revealed that impaired CFR remained an independent predictor (adjusted HR, 1.7 [95% CI 1.1–2.5]; p = 0.017).

**Table 3:** Univariate Cox proportional hazard regression analysis for prediction of MACE

Predictor	Univariate	
	HR (95% CI)	p value
Male	1.6 (1.2–2.1)	0.003
Age (per decade)	1.5 (1.3–1.7)	< 0.001
Hypertension	1.6 (1.2–2.2)	0.003
Dyslipidemia	1.4 (1.1–1.8)	0.021
Diabetes	1.5 (1.2–2.0)	0.001
Smoking	1.8 (1.4–2.4)	< 0.001
Family history of CAD	1.4 (1.0–1.9)	0.066
LVEF < 50%	2.4 (1.8–3.2)	< 0.001
Impaired CFR	2.3 (1.7–3.0)	< 0.001
Ischemia	2.4 (1.8–3.1)	< 0.001
LGE	2.6 (2.0–3.5)	< 0.001

HR: hazard ratio; CI: confidence interval; and other abbreviations as in Table 2

**Table 4:** Multivariate Cox proportional hazard regression analysis for prediction of MACE

Predictor	Multivariate	
	HR (95% CI)	p value
Male	1.0 (0.7–1.4)	0.964
Age (per decade)	1.4 (1.2–1.6)	< 0.001
Hypertension	1.2 (0.9–1.7)	0.249
Dyslipidemia	0.9 (0.7–1.3)	0.734
Diabetes	1.2 (0.9–1.6)	0.140
Smoking	1.4 (1.0–2.0)	0.029
LVEF < 50%	1.2 (1.2–2.3)	0.002
Impaired CFR	1.6 (1.2–2.1)	0.001
Ischemia	1.6 (1.2–2.1)	0.002
LGE	1.6 (1.1–2.1)	0.006

HR: hazard ratio; CI: confidence interval; and other abbreviations as in Table 2

**Fig. 6:**
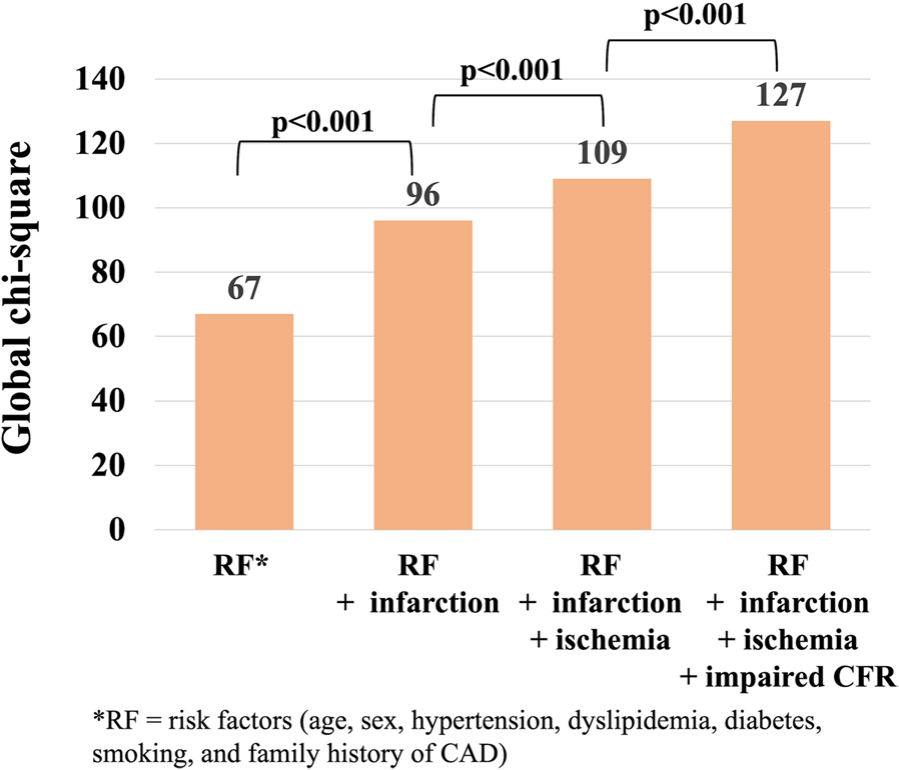
Incremental value of global CFR. Incremental prognostic value of global CFR over coronary risk factors and ischemia is shown. Adding infarction to coronary risk factors (male, increased age, hypertension, dyslipidemia, diabetes, smoking, and family history of coronary artery disease) increased global chi-square values from 67 to 96 (p < 0.001), and then, by adding ischemia to coronary risk factors plus infarction, global chi-square values increased to 109 (p < 0.001). Further, a global chi-square value of 127 was achieved by adding global CFR on top of coronary risk factors + infarction + ischemia (p < 0.001)

Adding prior MI to coronary risk factors (male, increased age, hypertension, dyslipidemia, diabetes, smoking, and family history of CAD) increased global chi-square values from 67 to 96 (p < 0.001), and then, by adding ischemia to coronary risk factors plus infarction, global chi-square values increased to 109 (p < 0.001). Further, a global chi-square value of 127 was achieved by adding global CFR on top of coronary risk factors + infarction + ischemia (p < 0.001; Fig. 6). Continuous NRI and integrated discrimination with the addition of global CFR to coronary risk factors plus ischemia improved to 0.352 (p < 0.001) and 0.017 (p < 0.001), respectively.

The Bland–Altman plot (Additional file 1: Fig. S5) summarized interobserver agreement in the 40 cases reviewed by the two experts and showed no bias (0.06) and systematic error with acceptable limits of agreement (-0.41–0.53). ICC was 0.98 (95% CI 0.96–0.99) and the interobserver agreement for detection of CFR tertiles was good (weighted kappa, 0.91; 95% CI 0.82–0.99).

## Discussion

This is the first study to evaluate the long-term prognostic value of CMR-derived CFR. The results can be summarized as follows: (1) in long-term follow-up, global CFR derived from the CS flow measurement provided a strong predictor of cardiovascular events; (2) global CFR and stress perfusion CMR provided complementary prognostic information to improve risk stratification; and (3) for both patients with and without myocardial ischemia, impaired global CFR was associated with worse prognosis, and the two subgroups showing disagreement between ischemia and impaired CFR had comparable prognoses.

Previous studies have demonstrated that global CFR as measured by positron emission tomography (PET) has excellent prognostic value in predicting MACE and cardiac death in patients with known or suspected CAD. Ziadi et al. [11] investigated the prognostic value of global CFR by ^82^Rb-PET in 677 patients using cardiac death and MI as primary endpoints. For both patients with and without abnormal relative perfusion, patients with impaired CFR (< 2.0) displayed poorer prognosis than those with preserved CFR. A more recent study by Murthy et al. [12] showed that, among 2,783 patients referred for rest and stress ^82^Rb-PET, the lowest tertile of CFR (< 1.5) was associated with a 5.6-fold increase in the risk of cardiac death compared with the highest tertile. PET has the advantage of regional measurement by CFR over CS-derived CFR, but requires ionizing radiation and an on-site ^82^Rb generator. Global CFR measurement in CS using CMR is easy to perform with short breath hold (15 s) while needing no ionizing radiation, contrast medium, or high-spec scanner. Further, for the post-processing of rest and stress images, it takes less than 10 min in total.

Several studies have investigated the prognostic value of global CFR by CS flow measurement. Kato et al. [6] evaluated the prognostic value of CS-derived global CFR in patients with suspected or known CAD during follow-up (median, 2.3 years) and showed that impaired global CFR was associated with higher risk of cardiac events in patients with known (HR, 5.17) or suspected (HR, 14.16) CAD. More recently, Indorkar et al. [7] demonstrated that, over a median follow-up of 2.1 years, CS-derived CFR provides prognostic information incremental to common clinical and CMR risk factors and emphasized the role of CS-derived CFR in identifying patients at risk of adverse events, particularly among patients with normal stress perfusion. Despite such promising results, those prior studies were limited by short durations of follow-up. Since CAD progression is slow, short-term follow-up is insufficient to assess the prognosis for CAD patients. The present long-term cohort with a larger study population showed that global CFR and stress perfusion CMR offered complementary prognostic information and, importantly, demonstrated an additive prognostic value of global CFR not only in patients without ischemia, but also in those with ischemia.

Global CFR is affected by epicardial coronary stenosis, diffuse coronary atherosclerosis, and microvascular dysfunction. The effect of global CFR on prognosis therefore depends on complex factors according to macro- or microvascular status. Since patients with impaired CFR in the absence of epicardial coronary stenosis have worse prognosis [13], diffuse atherosclerosis and microvascular dysfunction may represent essential influences on the relationship between impaired CFR and unfavorable prognosis. Diffuse atherosclerosis is reportedly associated with microvascular dysfunction, but this association is relatively weak [14, 15]. Importantly, a study by Naya et al. showed that the presence of microvascular dysfunction is more important for predicting cardiac events compared with diffuse atherosclerosis [15].

Several studies have investigated the relationship between global CFR and treatment for epicardial CAD. A prospective, multi-center observational study by Aikawa et al. evaluated CFR measured by ^15^O-water PET at baseline and 6 months after coronary revascularization, showing that the degree of improvement in angiographic CAD burden by revascularization correlated significantly with the magnitude of improvement in CFR [16]. This suggested that treatment for epicardial CAD could contribute to improvements in impaired CFR. In addition, a study by Taqueti et al. using ^82^Rb-PET investigated the effect of revascularization among patients with impaired or preserved CFR and revealed that, compared with individuals treated using optimal medical therapy, those who underwent coronary artery bypass grafting experienced better prognosis among patients with impaired CFR, but not among patients with preserved CFR [17]. Considering that the presence of both ischemia and impaired CFR was associated with the highest rates of events in the present study, the patient group with impaired CFR as well as ischemia may derive greater prognostic benefit from aggressive therapy including coronary revascularization. The presence of both ischemia and impaired CFR might thus provide a basis for identifying high-risk patients who should be prioritized for invasive or more intensive medical therapy. Future studies are warranted to test whether such a therapeutic strategy will improve the prognosis of patients with ischemia and impaired CFR.

This study showed disagreement between ischemia and impaired CFR in some patients. Impaired CFR without ischemia may be attributable to microvascular dysfunction or diffuse atherosclerosis rather than epicardial luminal narrowing. Similarly to the study by Indorkar et al. [7], the present study showed that, in the absence of ischemia, the prognosis of subjects with impaired CFR was worse than that in subjects with preserved CFR. Importantly, the subgroup with ischemia and no impaired CFR and the subgroup with no ischemia and impaired CFR displayed comparable prognoses. This finding suggests that microvascular dysfunction or diffuse atherosclerosis shown by impaired global CFR may be as important as ischemia due to epicardial luminal narrowing in the management and risk stratification of CAD patients. Furthermore, this study revealed that impaired CFR was associated with worse prognosis among patients who had no ischemia or infarction. Thus, even in the absence of ischemia or MI, patients with impaired CFR may require closer follow-up and more aggressive risk factor modification than those with preserved global CFR.

### Limitations

The study has several limitations. First, this was a single-center, retrospective study. A prospective multi-center study is warranted to confirm the results of this study. Second, this study used both 1.5- and 3T CMR scanners, which differed in several respects. It is of importance to note that the CMR parameters between 1.5T and 3T were not consistent, but when the analyses were performed for 1.5T and 3T separately, the results were not significantly different between 1.5T and 3T and were similar to those of the overall analysis. Third, the present study did not use quantitative perfusion mapping by CMR, which also allows assessment of both local and global CFR. Quantitative stress perfusion CMR might be able to show results similar to those of this study in a single test. However, stress perfusion CMR during the study period did not include quantitative method. The prognostic value of quantitative stress perfusion CMR may be a subject for future research.

## Conclusions

During long-term follow-up, global CFR derived from CS flow measurement provided additive and complementary prognostic value to stress perfusion CMR for the prediction of MACE and enhanced risk stratification for patients both with and without myocardial ischemia. The presence of both ischemia and impaired CFR, which was associated with the highest rates of events, might serve as a basis for selecting patients to undergo aggressive therapy, including coronary revascularization. Importantly, the prognoses of the subgroup with ischemia and no impaired CFR and the subgroup with no ischemia and impaired CFR were comparable. This implies that microvascular dysfunction or diffuse atherosclerosis shown by impaired global CFR may play a role as important as that of ischemia due to epicardial coronary stenosis in the management and risk stratification of CAD patients.

## Supplementary Information

**Additional file 1: Figure S1.** Long-term risk stratification for MACE in patients with known CAD. **Figure S2.** Long-term risk stratification for MACE in patients with suspected CAD. **Figure S3.** Long-term risk stratification for MACE in patients who underwent 1.5T CMR. **Figure S4.** Long-term risk stratification for MACE in patients who underwent 3T CMR. **Figure S5.** Bland–Altman plot for CFR measurement. **Table S1.** Cox proportional hazard regression analysis for prediction of MACE in patients with known CAD. **Table S2.** Cox proportional hazard regression analysis for prediction of MACE in patients with suspected CAD.

## Data Availability

Not applicable.

## Abbreviations

bSSFP: Balanced steady-state-free precession
CAD: Coronary artery disease
CFR: Coronary flow reserve
CI: Confidence interval
CMR: Cardiovascular magnetic resonance
CS: Coronary sinus
ECG: Electrocardiogram
FA: Flip angle
FOV: Fiel- of-view
HR: Hazard ratio
LGE: Late gadolinium enhancement
LV: Left ventricle/left ventricular
LVEDV: Left ventricular end-diastolic volume
LVEF: Left ventricular ejection fraction
LVESV: Left ventricular end-systolic volume
MACE: Major adverse cardiac events
MI: Myocardial infarction
NRI: Net reclassification improvement
PET: Positron emission tomography
TR: Repetition time
TE: Echo time
TFE: Turbo field echo
