# Supplementary material for: Complementary prognostic value of stress perfusion imaging and global coronary flow reserve derived from cardiovascular magnetic resonance: a long-term cohort study
Source: J Cardiovasc Magn Reson. 2023 Mar 16;25:20. doi: 10.1186/s12968-023-00930-3 (PMC10018821; doi:10.1186/s12968-023-00930-3)
Supplement: Supplementary file 1 — Additional file 1: Figure S1. Long-term risk stratification for MACE in patients with known CAD. Figure S2. Long-term risk stratification for MACE in patients with suspected CAD. Figure S3. Long-term risk stratification for MACE in patients who underwent 1.5T CMR. Figure S4. Long-term risk stratification for MACE in patients who underwent 3T CMR. Figure S5. Bland–Altman plot for CFR measurement. Table S1. Cox proportional hazard regression analysis for prediction of MACE in patients with known CAD. Table S2. Cox proportional hazard regression analysis for prediction of MACE in patients with suspected CAD. [file 12968_2023_930_MOESM1_ESM.docx]

The following figures and tables are Additional file for “**Complementary prognostic value of stress perfusion imaging and global coronary flow reserve derived from cardiovascular magnetic resonance: A long-term cohort study**” in ***Journal of Cardiovascular Magnetic Resonance***.

------------------------------------------------------------------------------------------------------------------------

**Figure S1. Long-term risk stratification for MACE in patients with known CAD**

The figures show that, in patients with known CAD, Kaplan-Meier curve analysis showed a significant difference in event-free survival among patients stratified by global CFR tertiles for prediction of MACE (log-rank, p=0.017; Figure-A), whereas there was not a significant difference in event-free survival among patients stratified by ischemia (Figure-B) or combination of impaired CFR and ischemia (Figure-C).

CAD = coronary artery disease; CFR = coronary flow reserve; CMR = cardiovascular magnetic resonance; and MACE = major adverse cardiac events.

**Figure S2. Long-term risk stratification for MACE in patients with suspected CAD**

The figures show that, in patients with suspected CAD, Kaplan-Meier curve analysis demonstrated a significant difference in event-free survival for prediction of MACE among patients stratified by global CFR tertiles (log-rank, p<0.001; Figure-A), ischemia (p<0.001; Figure-B) or combination of impaired CFR and ischemia (p<0.001; Figure-C).

CAD = coronary artery disease; CFR = coronary flow reserve; CMR = cardiovascular magnetic resonance; and MACE = major adverse cardiac events.

**Figure S3. Long-term risk stratification for MACE in patients who underwent 1.5T CMR**

The figures show that, among the patients who underwent 1.5T, Kaplan-Meier curve analysis demonstrated a significant difference in event-free survival for prediction of MACE among patients stratified by global CFR tertiles (log-rank, p<0.001; Figure-A), ischemia (p<0.001; Figure-B) or combination of impaired CFR and ischemia (p<0.001; Figure-C).

CFR = coronary flow reserve; CMR = cardiovascular magnetic resonance; and MACE = major adverse cardiac events.

**Figure S4. Long-term risk stratification for MACE in patients who underwent 3T CMR**

The figures show that, among the patients who underwent 3T, Kaplan-Meier curve analysis demonstrated a significant difference in event-free survival for prediction of MACE among patients stratified by global CFR tertiles (log-rank, p<0.001; Figure-A), ischemia (p<0.001; Figure-B) or combination of impaired CFR and ischemia (p<0.001; Figure-C).

CFR = coronary flow reserve; CMR = cardiovascular magnetic resonance; and MACE = major adverse cardiac events.

**Figure S5. Bland-altman plot for CFR measurement.**

The Bland-Altman plot showed no bias (0.06) and systematic error with acceptable limits of agreement (-0.41-0.53).

CFR = coronary flow reserve.

**Table S1.**

**Cox proportional hazard regression analysis for prediction of MACE in patients with known CAD**

| **Predictor** | **univariate** | | **multivariate** | |
| --- | --- | --- | --- | --- |
|  | **HR (95%CI)** | **p value** | **HR (95%CI)** | **p value** |
| Male | 1.4 (0.8-2.3) | 0.228 |  |  |
| Age (per decade) | 1.2 (1.0-1.5) | 0.035 | 1.2 (0.9-1.5) | 0.057 |
| Hypertension | 1.1 (0.7-1.8) | 0.635 |  |  |
| Dyslipidemia | 1.0 (0.6-1.5) | 0.866 |  |  |
| Diabetes | 1.2 (0.8-1.8) | 0.305 |  |  |
| Smoking | 1.3 (0.9-1.9) | 0.199 |  |  |
| Family history of CAD | 1.1 (0.7-1.7) | 0.689 |  |  |
| LVEF<50% | 1.8 (1.3-2.7) | 0.002 | 1.7 (1.2-2.5) | 0.004 |
| Impaired CFR | 1.6 (1.1-2.3) | 0.016 | 1.5 (1.0-2.1) | 0.048 |
| Ischemia | 1.2 (0.8-1.7) | 0.386 |  |  |
| LGE | 1.6 (0.9-2.6) | 0.066 |  |  |

CAD = coronary artery disease; CFR = coronary flow reserve; LGE = late gadolinium enhancement; LVEF = left ventricular ejection fraction; and MACE = major adverse cardiac events.

**Table S2.**

**Cox proportional hazard regression analysis for prediction of MACE in patients with suspected CAD**

| **Predictor** | **univariate** | | **multivariate** | |
| --- | --- | --- | --- | --- |
|  | **HR (95%CI)** | **p value** | **HR (95%CI)** | **p value** |
| Male | 1.3 (0.9-2.0) | 0.139 |  |  |
| Age (per decade) | 1.7 (1.3-2.1) | <0.001 | 1.4 (1.1-1.8) | 0.002 |
| Hypertension | 1.6 (1.0-2.4) | 0.031 | 1.2 (0.8-1.0) | 0.367 |
| Dyslipidemia | 1.26 (0.86-1.84) | 0.235 |  |  |
| Diabetes | 1.6 (1.1-2.4) | 0.016 | 1.2 (0.8-1.8) | 0.329 |
| Smoking | 2.0 (1.3-2.9) | <0.001 | 1.6 (1.1-2.4) | 0.014 |
| Family history of CAD | 1.4 (0.9-2.4) | 0.176 |  |  |
| LVEF<50% | 2.3 (1.4-3.8) | <0.001 | 1.6 (0.9-2.7) | 0.091 |
| Impaired CFR | 2.6 (1.8-3.8) | <0.001 | 1.7 (1.1-2.5) | 0.017 |
| Ischemia | 3.8 (2.6-5.6) | <0.001 | 2.4 (1.6-3.6) | <0.001 |
| LGE | 3.2 (2.1-4.7) | <0.001 | 1.8 (1.1-2.8) | 0.014 |

CAD = coronary artery disease; CFR = coronary flow reserve; LGE = late gadolinium enhancement; LVEF = left ventricular ejection fraction; and MACE = major adverse cardiac events.
